# The MAPK Pathway Signals Telomerase Modulation in Response to Isothiocyanate-Induced DNA Damage of Human Liver Cancer Cells

**DOI:** 10.1371/journal.pone.0053240

**Published:** 2013-01-31

**Authors:** Evelyn Lamy, Corinna Herz, Sabine Lutz-Bonengel, Anke Hertrampf, Melinda-Rita Márton, Volker Mersch-Sundermann

**Affiliations:** 1 Department of Environmental Health Sciences, University Medical Center Freiburg, Freiburg, Germany; 2 Institute of Legal Medicine, University Medical Center Freiburg, Freiburg, Germany; 3 Faculty of Biology, University of Freiburg, Freiburg, Germany; 4 Chemical Engineering Department, University Politehnica of Bucharest, Bucharest, Romania; H. Lee Moffitt Cancer Center & Research Institute, United States of America

## Abstract

4-methylthiobutyl isothiocyanate (MTBITC), an aliphatic, sulphuric compound from *Brassica* vegetables, possesses *in vitro* and *in vivo* antitumor activity. Recently we demonstrated the potent growth inhibitory potential of the DNA damaging agent MTBITC in human liver cancer cells. Here we now show that MTBITC down regulates telomerase which sensitizes cells to apoptosis induction. This is mediated by MAPK activation but independent from production of reactive oxygen species (ROS). Within one hour, MTBITC induced DNA damage in cancer cells correlating to a transient increase in hTERT mRNA expression which then turned into telomerase suppression, evident at mRNA as well as enzyme activity level. To clarify the role of MAPK for telomerase regulation, liver cancer cells were pre-treated with MAPK-specific inhibitors prior to MTBITC exposure. This clearly showed that transient elevation of hTERT mRNA expression was predominantly mediated by the MAPK family member JNK. In contrast, activated ERK1/2 and P38, but not JNK, signalled to telomerase abrogation and consequent apoptosis induction. DNA damage by MTBITC was also strongly abolished by MAPK inhibition. Oxidative stress, as analysed by DCF fluorescence assay, electron spin resonance spectroscopy and formation of 4-hydroxynonenal was found as not relevant for this process. Furthermore, N-acetylcysteine pre-treatment did not impact MTBITC-induced telomerase suppression or depolarization of the mitochondrial membrane potential as marker for apoptosis. Our data therefore imply that upon DNA damage by MTBITC, MAPK are essential for telomerase regulation and consequent growth impairment in liver tumor cells and this detail probably plays an important role in understanding the potential chemotherapeutic efficacy of ITC.

## Introduction

Telomerase provides a promising target for a *selective* therapeutic approach of malignancies in that 80 to 90% of cancer cells stably (re)express this enzyme while it is repressed in most normal somatic tissues [1]. hTERT, the catalytic subunit of the enzyme, is known to exert anti-apoptotic effects and interact with the DNA damage response pathway. In consequence cancer cells are more resistant against chemotherapeutic agents or radiation therapy [2], [3], [4], [5].

Isothiocyanates (ITC), naturally occurring secondary plant constituents of the family *Brassicaceae,* are known for their chemopreventive and -therapeutic actions both *in vitro* and *in vivo* [6], [7], [8]. A number of studies reported the growth suppressing and apoptosis inducing potency of this group in cancer cells and investigated underlying signalling pathways [9]. ITC have been shown to interfere with many factors that are altered in cancer cells such as interaction with the Bcl-2 family but they have also been shown to selectively decrease HDAC activity [10]. Recently ITC were shown as potent telomerase inhibitors during apoptosis induction in different cancer cells [11], [12], [13], [14]. Sulforaphane (SFN), e. g. suppressed telomerase during its proliferation inhibition of MCF-7 as well as MDA-MB-231 breast cancer cells [11]. Telomerase abrogation by SFN or phenylethyl ITC was also correlated with programmed death in HeLa cervical as well as PC-3 prostate cancer cells [13], [14]. SFN furthermore inhibited telomerase in human Hep3B liver cancer cells which paralleled programmed cell death [12]. This inhibition was then suggested to be mediated by production of reactive oxygen species (ROS). Other studies have demonstrated so far that oxidative stress and activation of the mitogen-activated (MAPK) signalling pathway were involved in the killing of cancer cells by ITC [15]. However, data published so far imply that ROS dependency of cell death as well as MAPK involvement might be cell specific.

In earlier studies, we already demonstrated the efficient growth impairment of liver cancer cells by ITC [16]. We thus aimed in the present study to investigate the relevance of MAPK activation and oxidative stress for cell death and telomerase regulation in human liver cancer cells. Therefore we used telomerase positive HCC cell lines (HepG2, Huh7 and Hep3B) differing in their tumor suppressor p53 (TP53) status as well as primary healthy human hepatocytes, devoid of telomerase. Our results confirm the activation of all three MAPK (JNK, ERK1/2 and P38) by MTBITC treatment independent from the TP53 or malignancy status of the cells. We could furthermore show that growth impairment as well as changes in telomerase level was signalled by MAPK but not related to ROS production. DNA damage triggered by MTBITC was inhibited in cells when MAPK were specifically blocked.

## Materials and Methods

### Chemicals

N-acetylcysteine (NAC), menadione, 2′, 7′ dichlorofluorescein diacetat (DCF-DA), dexamethasone, Tween® 20, benzo[a]pyrene (B(a)P and propidium iodide (PI) were acquired from Sigma Aldrich (Steinheim, Germany). DMSO (purity >99%) was from Applichem (Darmstadt, Germany). β-mercaptoethanol and valinomycin was purchased from Fluka (Buchs, Swiss). Dulbeccos Minimal Essential Medium (DMEM), fetal calf serum (FCS), trypsin 10× (25 mg/ml), trypsin-EDTA 10× (5 mg/ml, respectively 2.2 mg/ml), L-glutamine and phosphate buffered saline (PBS, without Ca and Mg) were from PAA Laboratories GmBH (Coelbe, Germany). Penicillin-Streptomycin (P/S) solution, RPMI-1640, 5,5′,6,6′-tetrachloro-1,1′,3,3′-tetraethylbenzimidazolcarbocyanine iodide (JC-1), insulin-transferrin-selen (ITS) and SYBR gold 10.000× were from life technologies Invitrogen (Darmstadt, Germany),. 4-Hydroxynonenal (HNE) was purchased from Cayman Europe (Tallinn, Estonia). 4-methylthiobutyl isothiocyanate (MTBITC, erucin) was synthesized by the Inst. of Organic Chemistry, University of Giessen, Germany as described before [17].

The p38 inhibitor SB203580, JNK inhibitor SP600125 and JNK inhibitor V were purchased from Santa Cruz, (California, USA). The ERK1/2 inhibitor U0126, p38 inhibitor SB202190 and ERK1/2 inhibitor PB98059 were obtained from Cell Signalling (Boston, USA). The following primary antibodies were used for immunoblotting: anti-p-ERK1/2 (Thr202/Tyr201, 1∶2000), anti-p-JNK (Thr 183/Tyr185, 1∶2000, clone G9), anti-p-c-Jun Ser73 and anti-p-p38 (Thr180/Tyr182, 1∶500) from Cell Signalling, anti- 4-Hydroxynonenal (1∶250; clone 198960) from R&D Systems EUROPE (Abingdon, England) and anti-beta-actin (1∶10000, clone AC-74) from Sigma Aldrich. The horseradish peroxidase (HRP)-labelled secondary antibodies anti-mouse and anti-rabbit were purchased from Cell Signalling. Nuclease free water was from Qiagen (Hilden, Germany). Caspase 3/7 GLO reagent was from Promega (Mannheim, Germany). MTBITC, menadione and the MAPK inhibitors were dissolved in sterile DMSO. HNE was dissolved in ethanol.

### HCC Cell Lines

HepG2 (wt-p53) and Hep3B (null-p53) cell lines were obtained from the German Collection of Microorganisms and Cell Cultures (DSMZ), Braunschweig, Germany. Huh-7 cells (mut-p53) originally established by Nakabayashi et al. [18] were kindly provided by H. Blum (University Medical Center Freiburg, Germany). The cells were cultured in low glucose DMEM supplemented with 15% (HepG2) or 10% (Huh7, Hep3B) FCS and 1% P/S in a 5% CO_2_ atmosphere at 37°C until 70% of confluency and subsequently harvested with trypsin. Cell line verification was done by microscopically checking the morphology of the cells and performing growth curve analysis on a regular basis. Only cells in passage number four to ten were used. The cell culture was furthermore tested negative for mycoplasma contamination.

### Primary Human Hepatocytes

Normal hepatocytes were isolated within 2 h from material taken from patients who had undergone partial hepatectomy and from whom prior written consent had been obtained. This part was approved by the ethics committee of the University of Freiburg. Assessment of non-tumorous liver parenchyma was performed by a senior pathologist with expertise in liver pathology. Hepatocytes were isolated using the two-step collagenase perfusion technique according to a modified protocol from Guguen-Guillouzo *et al.* [19] and Strom *et al.* [20]. The cells were then finally resuspended in RPMI-1640 supplemented with 15% FCS, 2 mM L-glutamine 1% P/S, 1% ITS, 100 nM dexamethasone and cultured in a 5% CO_2_ atmosphere at 37°C for 20 h.

### Determination of Drug Effect

Drug effect was tested at early passages (P4–P10). For the experiments, cells were seeded, supplemented with culture medium and incubated for 48 h at 37°C, 5% CO_2_ atmosphere. After that, the subconfluent cells were exposed to MTBITC for 1 to 48 h and subsequently processed for the assays. In some experiments, subconfluent cells were pre-treated with the antioxidant N-acetylcysteine (NAC) at concentrations between 1.25 to 10 mM for 1 h. Then the cells were thoroughly washed with PBS before adding MTBITC for another 24 h and subsequent assay preparation. In other experiments, subconfluent cells were pre-treated for one hour with specific MAPK inhibitors, either 10 µM SB203580, 2.5 µM or 5 µM SP600125 or 40 µM PB98059 or pre-treated for 2 h with 10 µM U0126, 20 µM JNK inhibitor V or 20 µM SB202190. A combination of inhibitors was also tested. Then the cells were thoroughly washed with PBS before adding MTBITC or 0.1% DMSO as solvent control and subsequent assay preparation.

### Single Cell Gel Electrophoresis (SCGE) Assay

The SCGE assay also known as comet assay was carried out as described earlier [21]. The % Tail DNA was calculated as indicator of DNA damage. For each sample, 102 systematically screened cells were evaluated.

### SubG1 DNA Content and Cell Cycle Distribution

Cellular DNA content was measured after permeabilization of harvested HCC cells by fixation with 70% ice cold ethanol for at least 24 h and staining of the DNA with PI master mix (40 µg/ml propidium iodide, 100 µg/ml Rnase (DNase free), PBS), allowing for the analysis of a DNA content frequency histogram. Analysis was performed by flow cytometry using a FACSCalibur (BD). The Modfit^©^ software was used to deconvolute the histograms to estimate the proportions of cells in particular phases of the cell cycle; the Cell Quest Pro^©^ software and FlowJo software (Treestar Inc, Ashlandd, USA) was used to quantify the percentage of apoptotic cells in the “sub-G1” or “hypoploid” peak.

### Caspase 3/7 Cleavage Assay

Caspase 3/7 cleavage was used as specific parameter for apoptosis induction. This was determined in HepG2 cells by using the Caspase3/7-Glo assay (Promega, Germany) according to the manufacturer’s instructions.

### Assessment of the Mitochondrial Membrane Potential (MMP)

For detection of changes in the MMP at the single cell level, the lipophilic cation JC-1 was used. Treated samples were harvested by trypsination, washed twice with PBS and resuspended in 1 ml of culture medium supplemented with the probe JC-1 at a final concentration of 2.5 µg/ml. The samples were resuspended and incubated under cell culture conditions for 30 min. Before analysis, the cells were washed twice with cold PBS and then directly analysed by a FACSCalibur (BD, Heidelberg, Germany).

### Detection of Cell Senescence by ß-galactosidase Staining

Senescence was detected using the senescence ß-galactosidase staining kit (Cell Signaling Technology, Boston, USA) according the manufacturer’s instructions. After cell treatment with MTBITC or controls pictures were captured using a bright light microscope (compact microscope system 8100E, Keyence, Osaka, Japan) with an objective Plan Apo 4×/0.2 and an optical magnification of 4× (Nikon, Japan).

### Protein Analysis by Immunoblotting

Proteins were analysed by immunoblotting after a modified protocol of Burnette [22]. Protein concentration was determined as described by Bradford [23]. For immunoblotting, 20 µg of protein were mixed with ready made SDS-containing sample buffer, supplemented with 1% beta-mercaptoethanol. Electrophoresis was performed according to the method of Laemmli [24]. The gel was then transferred to a nitrocellulose membrane by wet blotting (0.7 mA/cm^2^, 90 min.), blocked with 5% low fat milk in TBS/Tween 0.1% and incubated with primary antibody for 1 h at RT or over night at 4°C, and subsequently horseradish peroxidase (HRP)-labeled secondary antibody for 1 h at RT, each. After antibody incubation, the proteins of interest were detected by chemiluminescence technique. A digital image of the western blot was captured by the gel documentation system Molecular Imager® ChemiDoc™ XRS sytem (Bio-Rad, Munich, Germany). Size approximations were taken by comparing the stained bands to that of a protein standard loaded during electrophoresis. The process was repeated for the structural protein beta-actin. The amount of target protein could then be indexed to the structural protein to control between groups.

### Telomerase Activity Measurement by TRAP-Assay

Telomerase activity was detected using the TeloTAGGG ELISA Kit, commercially available from Roche (Mannheim, Germany). The assay was carried out after the manufacturerś instructions with slight modifications. For whole protein lysates cells were lysed, using cell lytic reagent from Sigma Aldrich containing 10 mM PSMF and 5 mM beta-mercaptoethanol for 30 min at 4°C. Protein concentration was determined after the method described by Bradford [23]. For telomerase reaction, a total reaction volume of 50 µl with equal amounts of protein and 2×TeloTAGGG reaction mix was incubated at 25°C for 20 min followed by denaturation at 94°C, 5 min, 30 cycles (at 94°C for 30 s, at 50°C for 30 s, and at 72°C, 90 s). Final elongation was carried out at 72°C, 10 min. As negative controls, an equal volume of lysis solution, nuclease free water and heat inactivated 0.1% DMSO treated sample (95°C for 5 min) were used. 5 µl or 2.5 µl PCR products were used for ELISA according manufacture’s protocol. 30 µl of the PCR products were loaded with 1×loading buffer on a TBE/12.5% polyacrylamid gel; electrophoresis was performed at 50 V for 30 min and subsequent 180 V for 5–6 h. The gel was stained with 1× SYBR gold/TBE solution for 20 min and detected under UV light. For molecular weight determination, 1 µg of 50 bp DNA ladder (Fermentas, St. Leon-Rot, Germany) was used.

### Quantitative Real Time-PCR of Full Length hTERT Transcript

Total RNA was isolated with the RNeasy mini Isolation kit from Qiagen (Hilden, Germany) followed by a purification step using the RNase-free DNase kit from Qiagen. Reverse transcription of 1 µg total RNA from each sample was performed in 20 µl using the First Strand cDNA Synthesis Kit from Fermentas. A 168 bp fragment of hTERT cDNA was amplified using the following oligonucleotides at the indicated end concentration: sense 5′GACACCATCCCCCAGGAĆ3 (50 nM) and antisense 5′TCGTGGAATGTTACGAGCAG3 (50 nM). Quantitative real time (qRT-)-PCR reaction was performed in a total volume of 25 µl, containing Maxima SYBR Green qPCR Master Mix 2× (Fermentas), indicated concentration of forward and reverse primer and 2.5 µl cDNA using 96-well 0.2 ml thin-wall PCR plates and MyIQ Real time PCR System (Bio-Rad, München, Germany). The qRT-PCR conditions were: 95°C for 10 min, followed by 40 cycles of 95°C for 10 s, 54°C for 60 s. Data were collected during the elongation step. Afterwards a melting curve was carried out to verify the specificity of the primer pairs. To normalize the amounts, the reference gene PBDG with the following oligonucleotides was used: sense 5′AGGATGGGCAACTGTACCTG3 (150 nM) and antisense 5′TCGTGGAATGTTACGAGCAG3 (150 nM). A 116 bp fragment was produced. The comparative ct method was used to calculate relative amounts of hTERT mRNA [25]. Each qRT-PCR reaction was performed in triplicate.

### Detection of ROS Production by DCF-DA and Electron Spin Resonance Spectroscopy

For ROS detection using DCF-DA, MTBITC-exposed cells were harvested, resuspended in fresh culture medium and immediately incubated with DCF-DA at a concentration of 5 nM for 15 min. at 37°C in the dark. Then, DCF fluorescence was measured with a FACSCalibur.

For ROS detection using electron spin resonance (ESR) spectroscopy the high cell permeable spin probe 1-hydroxy-3-methoxy-carbonyl-2,2,5,5-tetramethylpyrolidine hydrochloride (CMH, Noxygen Science Transfer & Diagnostics GmbH, Elzach, Germany) and the electron spin resonance (ESR) spectroscope E-Scan equipped with temperature and gas controller BioIII (Noxygen) was used. Adhered growing cells were incubated for 1 to 6 h with MTBITC, 100 µM menadione or 0.1% DMSO in culture medium. Afterwards, cells were washed once with Krebs-HEPES buffer supplemented with 25 µM deferoxamine (DFO) and 5 µM diethyldithiocarbonate (DETC, Noxygen) and incubated with 100 µM CMH spin probe in Krebs-HEPES buffer for 15 min at 37°C. Supernatants were transferred in new reaction tubes and kept on ice. ESR spectra were measured in 50 µl glass capillaries. Following instrument setting were used: microwave frequency 9.772GHZ and power 21.120 mW, receiver gain 1.00e+003, phase 0.20 deg, harmonic and modified amplitude 2.32 G, signal channel conversion 10.240 ms, time constant 40.960 ms and sweep time 5.24 s. The number of scans was 5 to 20.

### Flow Cytometry and Deposition of Single Cells

Single cells (HepG2) were deposited in individual wells of 96-well plates (Thermo Scientific, Bonn, Germany) using a MoFlo cell sorter (Dako, Glostrup, Denmark). The cells were sorted using forward (low angle, FSC) versus side scatter (90°-scatter, SSC) signals to distinguish between live and dead cells and debris and the area of forward scatter versus pulse width to distinguish between single cells and cells attached to each other. No cells were placed on row 12, and these spots were used for 4 PCR positive and 4 negative controls, respectively.

### Treatment of the Cells with UV-irradiation

The uncovered 96 well plates containing single cells were subjected to UV irradiation for 10 min. using a UV decontamination chamber. The UV source was a 30 watt Osram germicidal lamp (UVC radiated power 200–280 nm 13.4 W). The efficiency of UV-irradiation to destroy double-stranded DNA was assayed by amplifying DNA fragments of four sizes from 143 bp to 1337 bp.

### mtDNA Amplification

Amplification of mtDNA from treated and untreated cells was carried out in 96-well plates as received from the single cell deposition technique. To each well 5 µl of PCR master mix containing 1×Advantage 2 PCR buffer (Clontech, BD Biosciences, CA, USA), 0.1 µl of Advantage 2 polymerase mix (Clontech), 200 µM each dNTP (Bioline, Luckenwalde, Germany) and 0,4 µM each primer (F14816/R16152, F16197/R00293, F00314/R00639 and F08294/R08436, cf. Table 1) (Biomers, Ulm, Germany) were added. PCR amplification was performed on a PTC 200 thermal cycler (MJ Research, Waltham, MA, USA) with the following thermal cycling conditions: 95°C for 2 min, 38 cycles of 95°C for 15 s, 56°C for 30 s, 72°C for 90 s, followed by a final extension phase at 72°C for 10 min. The whole reaction was visualized under standardized conditions on a 2% agarose gel (50 ml agarose with 4 µL SybrSafe (Invitrogen Carlsbad, CA), electrophoresis for 25 min at 60 V, followed by exposure to UV light for 160 msec).

**Table 1:** Sequences and fragment length of primer pairs used for mtDNA amplification.

Primer	Sequence (5′ to 3′)	Fragment length
F14816*	CCATCCAACATCTCAGCATGATGAAA	1337 bp
R16152**	AGGTGGTCAAGTATTTATGGTAC	
F16197**	CTTACAAGCAAGTACAGCAATCAAC	666 bp
R293**	AATTTTTTGTTATGATGTCTGTGTGG	
F314	CCGCTTCTGGCCACAGCACT	326 bp
R639	GGGTGATGTGAGCCCGTCTA	
F8294***	CCACTGTAAAGCTAACTTAGCATTAACC	143 bp
R8436***	GTGATGAGGAATAGTGTAAGGAGTATGG	

* according to: Parson et al. [37] ** according to: Eichmann et al. [38] *** according to: Andréasson et al. [39].

### Data Analysis

Results were analysed using Graphpad Prism 5 software (GraphPad Software Inc., LaJolla, USA). Statistical significance (p≤0.05, p≤0.01) of MTBITC treatment on the investigated parameters in HCC cells was calculated against the solvent control, 0.1% DMSO. To determine the significance of MAPK specific inhibition on the investigated endpoints, MTBITC-treated samples were calculated against the respective inhibitor-treated counterpart using the one-way ANOVA followed by Bonferroni correction.

## Results

### nDNA is Damaged by MTBITC which Triggers Transient hTERT mRNA Expression

**Figure 1:**
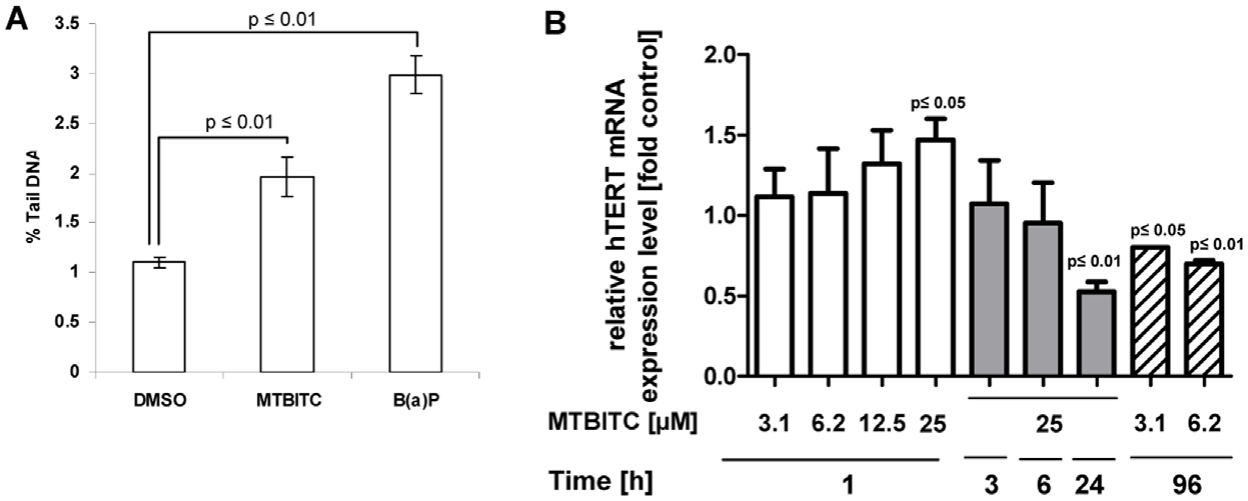
DNA damage leads to transient hTERT mRNA expression in HepG2 cells. **a)** Cells were exposed to 25 µM MTBITC or the solvent control (0.1% DMSO) for 1 h and subsequently analyzed for their DNA damage using the comet assay. The % tail DNA was used for damage quantification. Bars are mean ± SD, n = 3. Cells exposed to 100 µM benzo(a)pyrene (B(a)P) for 1 h were used as positive control. **b)** Expression of full length hTERT mRNA after exposure to MTBITC or solvent control for 1 h to 96 h was analysed by RT-PCR. PBDG was used in all experiments as reference gene. mRNA expression was calculated relative to the solvent control; bars are mean ± SD (n = 3).

We first investigated the damage of nDNA in HepG2 cells after 25 µM MTBITC exposure. After 1 h, nDNA of MTBITC-treated cells was significantly damaged, as determined by the comet assay (figure 1a). This was correlated with a fast response of cells in terms of transient hTERT mRNA expression as detected within 1 h (figure 1b). hTERT mRNA expression was back to control levels after 6 h exposure to MTBITC (figure 1b) and cell exposure to the ITC for more than 6 h resulted in a substantial suppression of hTERT mRNA transcription, which could also be observed at long-term low-dose exposure (figure1b).

### MTBITC Treatment Causes Activation of the MAPK Pathway

**Figure 2:**
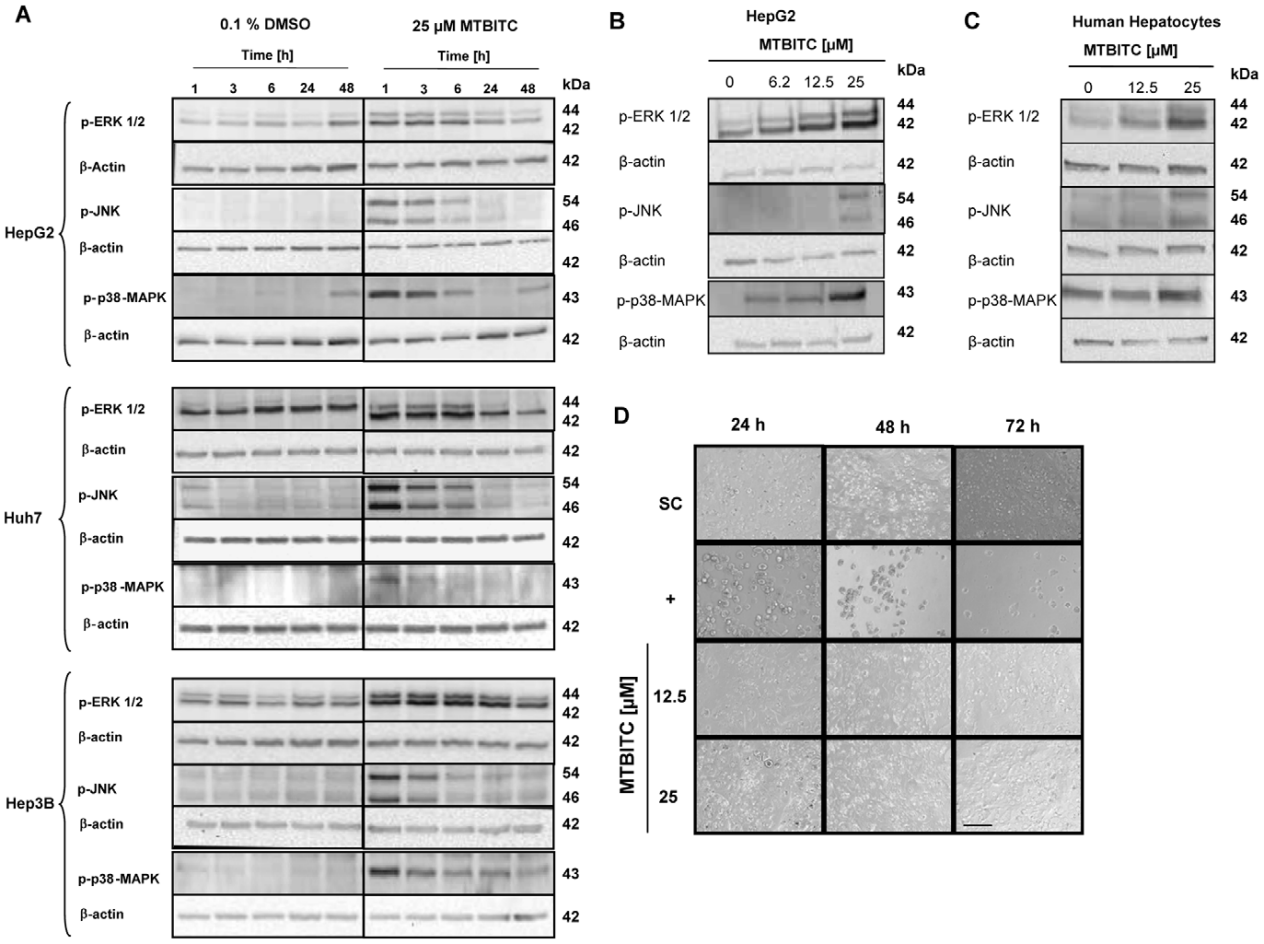
MAPK are activated upon MTBITC exposure. Cells were lysed and total lysate subjected to immunoblotting. **a)** HCC cells were treated with 25 µM MTBITC or the solvent control (0.1% DMSO) for the indicated time points. HepG2 cells (**b**) or human hepatocytes (**c**) were treated with MTBITC for 3 h at the indicated concentrations. Representative blots are shown. Each blot was reprobed with anti-β-actin antibody to ensure equal protein loading. **d)** Primary normal human hepatocytes were not adversely affected by MTBITC, as evident in representative pictures, captured by light microscopy. SC: solvent control = 0.1% DMSO. +, positive control = 0.01% triton X-100. Bar = 100 µm. All panels have the same magnification.

The MAPK signal transduction pathway is preferentially activated in response to environmental and genotoxic stresses and occupies a key role in cell proliferation and survival [26]. Growth impairment by ITC has been attributed to the activation of the MAPK pathway in different cell models before [27]. We therefore next investigated the response of MAPK in liver cancer cells to MTBTIC. We observed that in all three cell lines MTBITC treatment resulted in substantial activation of ERK1/2 at Thr202/Tyr204, JNK at Tyr183/Tyr185 and P38 at Thr180/Tyr182 by phosphorylation in a time- (figure 2a) but also concentration dependent manner (figure 2b). Interestingly, in healthy human hepatocytes devoid of telomerase, this pathway was also activated (figure 2c). To then determine whether this activation is reflected by cell impairment, we next addressed the viability of normal primary human hepatocytes. As shown in figure 2d, even after 72 h exposure to MTBITC, no adverse effect could be detected, as determined by microscopic observation of cell morphology.

### Role of MAPK in MTBITC-mediated Telomerase Regulation

**Figure 3:**
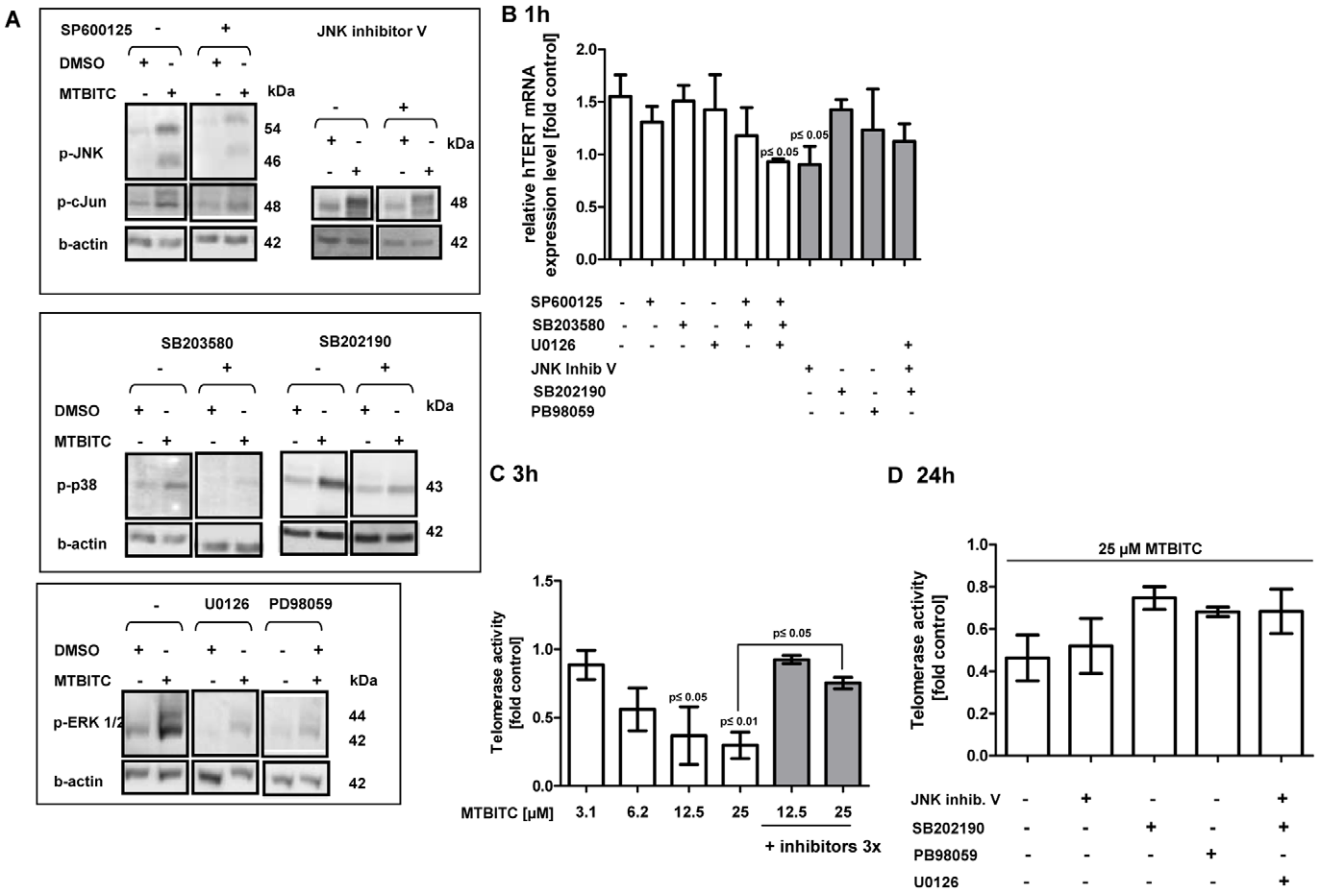
Effect of specific MAPK inhibitors on telomerase. For specific inhibition of p38 activation, 10 µM SB203580 or 20 µM SB202190, of JNK activation, 5 µM SP600125 or 20 µM JNK inhibitor V were used, respectively. 10 µM U0126 or 40 µM PB98059 were used for ERK1/2 inhibition. Cells were pre-treated with the inhibitors and subsequently exposed to MTBITC or the solvent control (0.1% DMSO). **a)** Immunoblot analysis shows effective inhibition of the target proteins. β-actin was used as loading control **b)** Pre-treated cells were analysed for full length hTERT mRNA expression after 1 h exposure to 25 µM MTBITC. PBDG was used as reference gene. mRNA expression was calculated relative to the corresponding solvent control (n = 3). **c+d)** Pre-treated cells were assayed for telomerase enzyme activity after 3 h (d) or 24 h (e) MTBITC exposure using the TRAP-ELISA assay. Telomerase activity was calculated relative to the corresponding solvent control; bars are mean ± SEM (n = 3). Inhibitors 3×: combination of SB203580, SP600125 and U0126.

To determine the relevance of the observed MAPK activation in telomerase modulation as well as cell proliferation, we pre-treated HepG2 cells with JNK inhibitor (SP600125 or JNK inhibitor V), P38 inhibitor (SB203580 or SB202190), ERK1/2 inhibitor (U0126 or PB98059) or a combination, followed by treatment with MTBITC. Activation of the MAPK through MTBITC was almost completely abolished by the pre-incubation with the inhibitors, as depicted in figure 3a. Pre-treatment of HepG2 cells with either of the inhibitors – except for JNK inhibitor V – failed to significantly affect MTBITC-triggered hTERT mRNA expression observed after 1 h (figure 3b). Interestingly, a combination of all three MAPK inhibitors (SP600125, SB203580, U0126 or SB202190, JNK inhibitor V and U0126) reduced MTBITC-triggered hTERT mRNA expression elevation (figure 3b). This may very well be due to inhibition of JNK activation, as either pre-treatment with SP600125 or JNK inhibitor V alone abolished hTERT mRNA level enhancement (figure 3b). Finally, we determined the relevance of MAPK for MTBITC-induced telomerase enzyme activity suppression. A concentration dependent downregulation of enzyme activity was already evident after 3 h exposure to MTBITC, as assessed by TRAP-ELISA (figure 3c). This could be clearly abolished by MAPK blocking. Moreover, by selectively blocking MAPK, we could show that p38 as well as ERK1/2 but not JNK were at least partly responsible for telomerase enzyme inhibition by MTBITC as assessed after 24 h exposure (figure 3d).

### Role of MAPK in MTBITC-mediated Growth Attenuation

**Figure 4:**
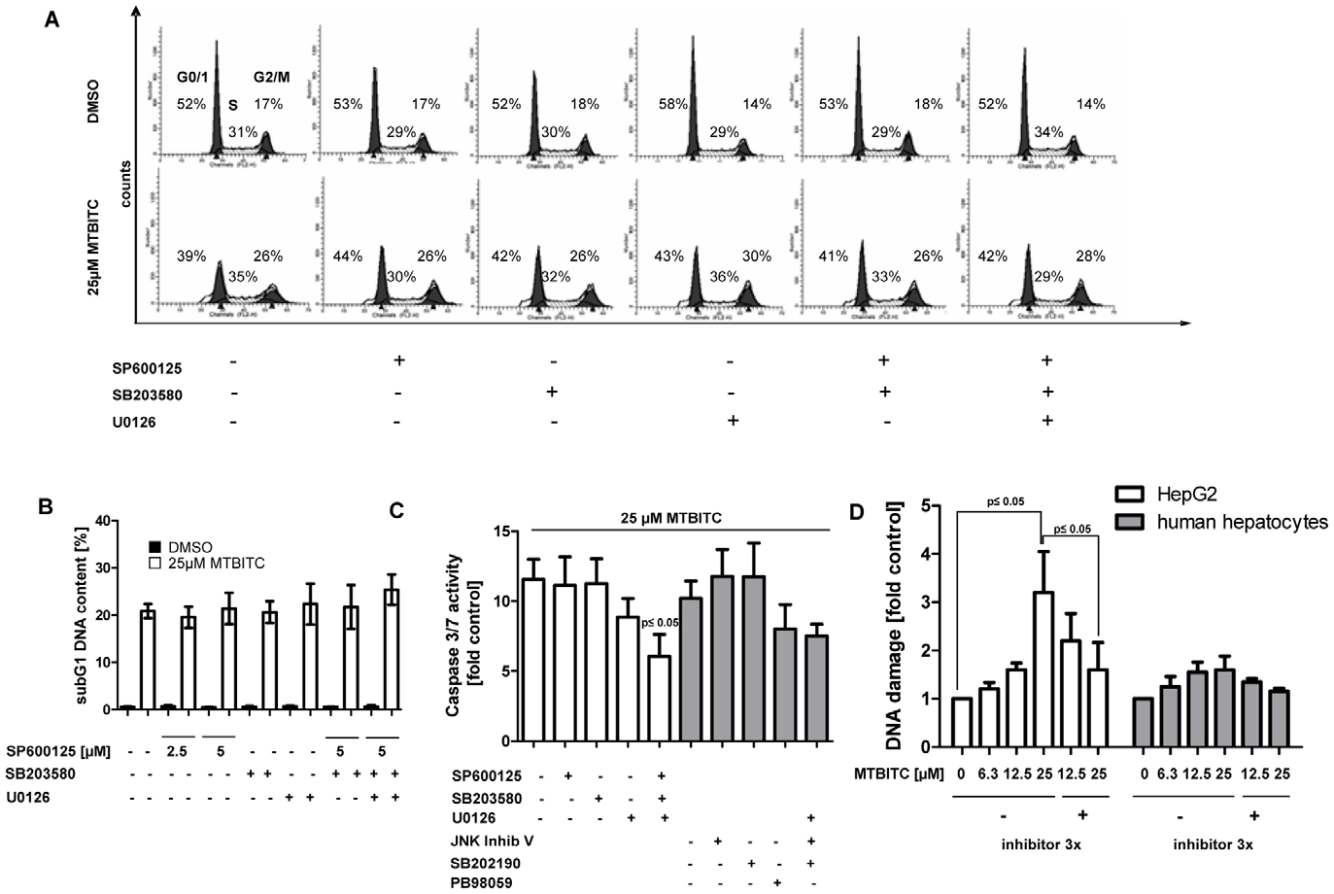
MAPK mediate DNA damage and apoptosis but not cell cycle arrest. Pre-treated HepG2 cells were exposed to 25 µM MTBITC or 0.1% DMSO for 24 h and then prepared for **a)** DNA content and **b)** subG1 DNA content analysis (marker for apoptosis) using flow cytometry and PI staining of DNA The pictures depict representative DNA content histograms of HepG2 cells. **c)** Caspase 3/7 cleavage was used as specific parameter for apoptosis induction. Caspase 3/7 activity was calculated relative to the corresponding solvent control; bars are mean ± SD (n = 3). **d)** DNA damage in HepG2 cells or primary human hepatocytes was assessed after 24 h exposure to MTBITC using the comet assay. To compare between the two cell types, DNA damage was calculated relative to the solvent control (SC, 0.1% DMSO). Bars are mean ± SD, n = 2. Inhibitors 3×: combination of SB203580, SP600125 and U0126.

In a next step, we sought to determine whether MAPK could also account for MTBITC-induced cell cycle arrest in HepG2 cells. However, all inhibitors as single component or in combination failed to protect the cells from MTBITC-mediated G2/M halt (figure 4a). This observation was confirmed by using a combination of the second inhibitor set (data not shown). When studying apoptosis by subG1 DNA content analysis, no relevance of MAPK for MTBITC-triggered cell death could be seen (figure 4b). However, quantification of apoptosis in terms of the more specific caspase 3/7 cleavage revealed that blocking of all three MAPK significantly abrogated MTBITC-triggered cell death. This could be attributed to ERK1/2 signaling, as inhibition of ERK1/2 activation strongly abolished apoptosis (figure 4b). To further establish the association of apoptosis and DNA damage with MAPK signaling, we quantified the effect of MAPK inhibitors on MTBITC-induced DNA strand breaks. As both, HCC cells as well as healthy hepatocytes were activated in their MAPK signaling by MTBITC, but only the cancer cells were submitted to apoptosis, we raised the question whether there could be a difference in the amount of DNA damage between the normal and malignant cells. As depicted in figure 4c, DNA strand breaks were indeed much lower in healthy hepatocyes as compared to HepG2 cells. When cells were then pre-treated with a combination of all three inhibitors, the damage was reduced to control level (figure 4c).

### ROS are not Involved in MTBITC-mediated Telomerase Modulation or Growth Impairment

**Figure 5:**
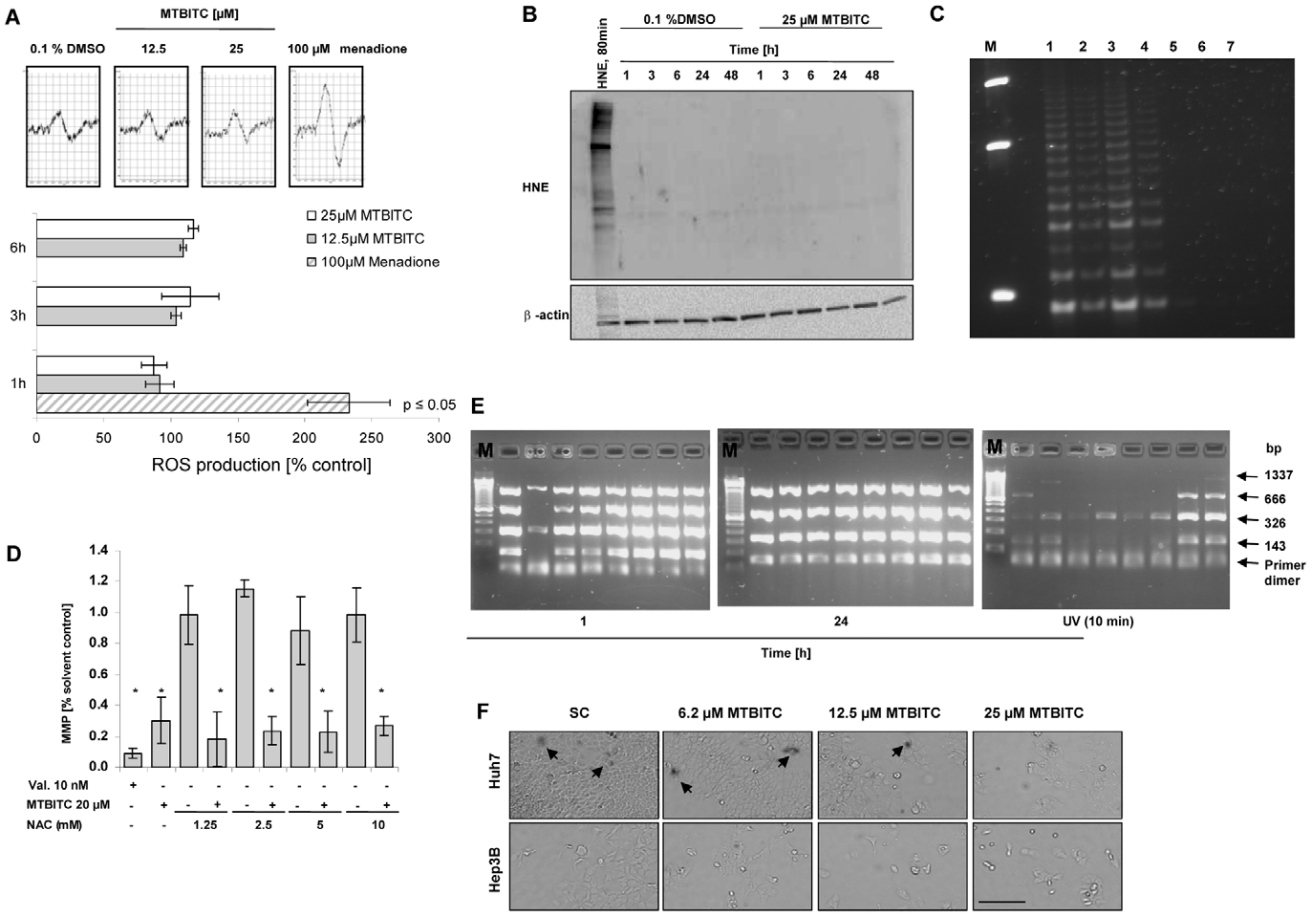
Apoptosis induction and telomerase loss in HepG2 cells are independent from ROS production. **a)** Effect of MTBITC on ROS production in adherent growing cells. Cells were exposed to MTBITC for 1 to 6 h, washed thoroughly with PBS and subsequently exposed to 100 µM spin probe CMH in Krebs-Hepes buffer. ROS were then quantified using ESR spectrometry. Positive control: 100 µM menadione. The pictures display the middle peak of ESR spectra of CMH spin probe labelled samples. Bars are mean ± SD, n = 3. **b)** Representative immunoblot for HNE Lys-adducts after exposure to MTBITC or DMSO for different time points. Total lysate of HepG2 cells was subjected to immunoblotting using an anti-HNE Lys antibody. Exposure of cells to HNE for 80 min. was used as positive control. The blot was reprobed with anti-β-actin antibody to ensure equal protein loading. **c+d)** Effect of NAC pre-treatment. HepG2 cells were pre-treated with NAC for 1 h, washed with PBS and subsequently exposed to 25 µM MTBITC or 0.1% DMSO for another 24 h. Cells were then prepared for (c) TRAP analysis or (d) flow cytometric analysis of the mitochondrial membrane potential (MMP) as parameter for apoptosis; *p≤0.05. M: 50 bp DNA marker; 1) 0.1% DMSO 2) 25 µM MTBITC 3) 5 mM NAC +0.1% DMSO,4) 5 mM NAC +25 µM MTBITC 5) cell lytic buffer 6) destilled water 7) 0.1% DMSO, heat treated. **e)** Effect of 25 µM MTBITC on mtDNA damage after 1 or 24 h exposure of HepG2 cells. Agarose gel electrophoresis of amplified mtDNA multiplex PCR products is shown in representative image details. Each lane contains amplification products obtained with mtDNA from one single cell using specific PCR primers. As positive control for mtDNA damage, cells were exposed to 10 min UV irradiation. f**)** Representative pictures of beta-galactosidase staining of HCC cells after exposure to MTBITC for 72 h, captured by light microscopy. beta-galactosidase positive cells are reflected by dark color of the cells. SC: solvent control = 0.1% DMSO. Scale bar = 200 µm, all panels have the same magnification.

Some other natural compounds have been proposed recently to mediate telomerase suppression in cancer cells via ROS production [28], [29]. Furthermore, a number of studies investigating ITC in the context of cancer cell growth suppression proposed ROS production as upstream event for MAPK activation and cell death and/or growth arrest [15]. In the context of our study, we therefore sought to find out whether ROS were involved in telomerase modulation by MTBITC or could act as cell death effectors. Intracellular ROS in control- and MTBITC-treated cells was first assessed by flow cytometry following staining of adherent growing HepG2 cells with H_2_DCFDA. Within one hour of MTBITC-exposure, no change in ROS level could be detected (data not shown). However, since the detection of ROS with this fluorescent probe entails many well known problems [30], we additionally used ESR in combination with the probe CMH for a specific quantification of intracellular as well as extracellular ROS. Representative diagrams of the spin signal for control and treated cells are depicted in figure 5a. As can be seen between 1 to 3 h, MTBITC-treated cells did not exhibit any significant change in mean CMH formation compared to control cells. Only after 6 h, a slight increase could be detected (figure 5a) which confirms earlier results by our group, generated with the fluorescent probe dihydrorhodamine 123 [16]. To verify these observations, we investigated the production of 4-hydroxynonenal (HNE). During oxidative stress, aldehyde by-products of lipoperoxidation, particularly HNE, are produced. HNE in turn is a known inhibitor of telomerase. This occurs by modulating Myc/Mad-1 transcription factor expression which in consequence decreases hTERT promotor activity [31], [32]. Probing cell lysates with HNE antibody, however, confirmed the absence of HNE adduct levels compared with control cells (figure 5b). Another experimental setup was then made using NAC pre-treatment of cells before MTBITC exposure. NAC is a well described antioxidant which is capable of restoring intracellular glutathione levels and scavenging ROS. As evident in figure 5c, NAC pre-treatment did not prevent the cells from MTBITC-mediated telomerase activity loss. Furthermore, NAC in a concentration range between 1.25 to 10 mM could not save the cells from MMP depolarization induced by MTBITC (figure 5d). Once damaged, mtDNA can amplify oxidative stress by decreased expression of critical proteins important for electron transport leading to a vicious cycle of ROS and organellar dysregulation that eventually trigger apoptosis [33]. We therefore additionally analysed whether MTBITC could also damage mtDNA besides nDNA then amplifying the signalling into cell death. But, as shown in figure 5e, no increase in mtDNA damage could be seen within 1 h and even not at a stage of mitochondrial depolarization (after 24 h). Exposure of cells to UV radiation, which was used as positive control, resulted in a clear degradation of different sized mitochondrial DNA.

Besides apoptosis induction, hTERT down regulation can result in premature cell senescence, as demonstrated earlier [34]. Furthermore, it was recently proposed that sulforaphane, a reduced form of MTBITC, induce both, senescence as well as apoptosis, in human mesenchymal stem cell cultures by modulation of DNA damage and inhibition of DNA repair genes [35], [36]. Therefore, in a last set of experiments we investigated whether MTBITC could also trigger HCC cells into a senescent state. These cells are typically characterized by a large morphology and expression of a senescence-associated beta-galactosidase. However, none of the investigated HCC cell lines were subjected to senescence by MTBITC, as analyzed after 72 h of exposure (figure 5f).

## Discussion

Stress, including genotoxic events, activates mitogen-stimulating signals like the MAPK/ERK signaling pathway. These in turn either phosphorylate cytoplasmic targets or translocate into the nucleus leading to the regulation of transcription factors involved in cell growth, survival or differentiation. While ERK1/2 activation has been associated with cell proliferation and survival, JNK and p38 are deemed stress responsive and thus involved in apoptosis [37]. In line with this, MAPK were identified as indirect stimulators or repressors of the hTERT promoter. So, activation of JNK in ovarian surface epithelial cells induced telomerase activity by driving the hTERT promoter. This was then suggested to be mediated by c-Jun activation [38]. Moreover, c-Jun phosphorylation by JNK is a common event triggered by DNA damage leading to either apoptosis or cell survival [39]. These processes concur with the observations made in our present study, where we could identify JNK and consequently c-Jun activation to be relevant for early transient hTERT mRNA expression upon MTBITC triggered DNA damage in HCC cells. Later on, MTBITC exposure resulted in telomerase inhibition which correlated with apoptosis induction. This was then presumably caused by the MAPK p38 as well as ERK1/2, as blocking of either one restored telomerase whereas JNK was found not relevant. Consequently, ERK1/2 inhibition partly abolished apoptosis induction in HepG2 cells. These findings suggest a connection between telomerase activity and cell survival capacity via MAPK. ERK1/2 as well as p38 were both demonstrated earlier to mediate the inhibition of hTERT transcription via c-Myc suppression [40], [41], [42]. The protooncogene c-Myc is an important transcription factor of hTERT, inducing hTERT transcription by binding to E-boxes in the promoter [43], [44], [45], [46]. More exactly, it has been found, that binding of the dimer complex Myc/Max to the E-box sequence enhanced hTERT expression [43], [45], [47], whereas a complex formed by Mad1 and Max repressed the promoter [45], [48]. c-Myc inhibition upon ITC exposure was already described in human breast cancer cells [11] and it could be possible that the effect of MTBITC observed on telomerase in the present study is also due to c-Myc inhibition but this needs to be investigated.

In contrast to HCC cells, primary human hepatocytes, although also activated in all three MAPK, were not killed by MTBITC which underlines the selective acting of ITC on liver cancer cells. The intensity of DNA damage induced by MTBITC was much weaker compared to HepG2 cells which could provide an explanation for the increased tolerance of the normal cells against the ITC. Interestingly, blocking of MAPK not only abolished the DNA damage in HCC cells but also in the healthy counterpart. Upon DNA damage, a variety of cellular mechanisms are activated to repair genomic lesions. In case damage is too extensive, a cellular suicide response/programmed cell death is induced. However, many differences exist between cancer and normal cells. These include that normal cells repair DNA damage more readily than their malignant counterpart. Additionally, great variability in DNA repair capacity is observed between different cell lines and telomerase activity is currently discussed to account for an altered repair of cancer cells [49]. The data by Zanichelli et al. [36] also imply a relevance of DNA repair in the response of normal stem cells to ITC. We therefore suggest to systematically explore the relevance of DNA damage network activation in the context of MTBITCś selectivity on cancer cells in future studies.

MAPK phosphorylation has often been linked to ROS production, which then could act as messengers in the intracellular signalling cascades [50]. ROS in turn have been reported to mediate telomerase suppression [28] which was also detected in MTBITC-exposed HCC cells at a later time point (>6 h) in the present study. But the capability of ITC to induce ROS is thought to be strictly cell type specific [51]. In human liver cancer cells, generation of oxidative stress was either found relevant for ITC cytotoxicity [12] or not [52]. In our study we applied a whole test battery consisting of specific ROS markers and antioxidants and could therefore ensure that ROS are not relevant for telomerase suppression or growth impairment of liver cancer cells by MTBITC. This concurs with an earlier study conducted by our group, which identified ROS by DHR123 as downstream event of ITC-induced mitochondrial membrane depolarization [16]. Breaks within the mtDNA are closely interrelated with mitochondrial ROS production but in the present study we could even exclude mtDNA damage as causal role for growth impairment.

### Conclusions

Intensity and duration of transient telomerase activation upon genotoxic stress are potential important factors for cancer cell protection against anti-neoplastic agents. Evidence that elevated telomerase activity could provide a survival advantage of cancer cells and presents another anti-apoptotic cell response upon DNA damage was already provided by several studies [53], [54], [55]. However, the exact mechanism as well as telomerase significance in cell cycle regulation and apoptosis induction in response to DNA damage by MTBITC still needs to be further defined. So far no study has investigated MAPK as upstream regulators of telomerase in direct response to a DNA damaging insult. Our data now demonstrate that MAPK, which are early activated by MTBITC-triggerd DNA damage, are indeed relevant for telomerase regulation in HCC cells. Additionally, telomerase is suggested to play a decisive role in protecting HepG2 cells against MTBITC, as telomerase restoration by MAPK inhibition concurred with a reduced DNA damage and consequently apoptosis induction. As most cancer cells are hTERT-positive, this could provide a very good reason to make efforts for understanding its regulation by ITC on the molecular level as well as its implications in cancer cell survival.

Furthermore, our findings underline the differential acting of MAPK on factors which orchestrate the cellular response to DNA damaging agents. This is in accordance to the concept of cell response that allows either cell adaption to the altered conditions or triggers programmed death and based on our data, telomerase is proposed to represent one determinant of a cancer cellś vulnerability to MTBITC-induced apoptosis.
