# Expression of Concern: The MAPK Pathway Signals Telomerase Modulation in Response to Isothiocyanate-Induced DNA Damage of Human Liver Cancer Cells

**DOI:** 10.1371/journal.pone.0344267

**Published:** 2026-03-09

**Authors:** The PLOS One Editors

Following the publication of this article [1], concerns were raised with results presented in Figs 2 and 3. Specifically,

- The Huh7 middle and bottom β-actin panels in Fig 2A appear similar to the Huh7 β-actin panels of Fig 6C of [2].
- The Hep3B bottom β-actin panels in Fig 2A appear similar to the Hep3B β-actin panels of Fig 6C of [2].
- The HepG2 bottom β-actin panels of Fig 2A appear similar to the HepG2 top β-actin panels in Fig 5E of [3].
- The Huh7 top β-actin panels of Fig 2A appear similar to the Huh7 top β-actin panels in Fig 5E of [3] and to the Hep3B β-actin panels in Fig 5D of [3].
- The SP6001125 β-actin panels of Fig 3A appear similar to the SB203580 β-actin panels of Fig 3A.
- The p-ERK 1/2 U0126 panel of Fig 3A appears similar to the p-ERK 1/2 PD98059 panel of Fig 3A.

The corresponding author stated that in order to allow direct comparability of the respective target protein signals with the corresponding β-actin loading control, β-actin loading controls were shown more than once in cases where the same membrane was used for the detection of different target proteins. They also stated that, in Figure 5D of [3], the labeling of the Huh7 and Hep3B samples was inadvertently interchanged. The corresponding author stated that the original raw data for [1] are no longer available.

In light of the above concerns which cannot be fully resolved in the absence of the underlying data, the *PLOS One* Editors issue this Expression of Concern.
